# Flavonoids as Putative Epi-Modulators: Insight into Their Binding Mode with BRD4 Bromodomains Using Molecular Docking and Dynamics

**DOI:** 10.3390/biom8030061

**Published:** 2018-07-23

**Authors:** Fernando D. Prieto-Martínez, José L. Medina-Franco

**Affiliations:** Facultad de Química, Departamento de Farmacia, Universidad Nacional Autónoma de México, Avenida Universidad 3000, Mexico City 04510, Mexico

**Keywords:** docking, epigenetics, epi-informatics, molecular interactions, molecular dynamics, natural products, flavonoids

## Abstract

Flavonoids are widely recognized as natural polydrugs, given their anti-inflammatory, antioxidant, sedative, and antineoplastic activities. Recently, different studies showed that flavonoids have the potential to inhibit bromodomain and extraterminal (BET) bromodomains. Previous reports suggested that flavonoids bind between the Z and A loops of the bromodomain (ZA channel) due to their orientation and interactions with P86, V87, L92, L94, and N140. Herein, a comprehensive characterization of the binding modes of fisetin and the biflavonoid, amentoflavone, is discussed. To this end, both compounds were docked with BET bromodomain 4 (BRD4) using four docking programs. The results were post-processed with protein–ligand interaction fingerprints. To gain further insight into the binding mode of the two natural products, the docking results were further analyzed with molecular dynamics simulations. The results showed that amentoflavone makes numerous contacts in the ZA channel, as previously described for flavonoids and kinase inhibitors. It was also found that amentoflavone can potentially make contacts with non-canonical residues for BET inhibition. Most of these contacts were not observed with fisetin. Based on these results, amentoflavone was experimentally tested for BRD4 inhibition, showing activity in the micromolar range. This work may serve as the basis for scaffold optimization and the further characterization of flavonoids as BET inhibitors.

## 1. Introduction

Epigenetics has arisen as the missing link in the biogenesis of disease. Histone modifications have a significant effect on the fate of certain genes. Current research is primarily focused on the writing and erasing mechanisms of the epigenome. There are many examples of this in the literature [1], one of the most prominent being histone acetylation. Acetylation is regulated by two main systems: histone acetyl transferases (HATs) and histone deacetylases (HDACs) [2]. Histone deacetylases have been studied thoroughly by means of pharmacophore modeling [3], molecular docking [4], and molecular dynamics (MD) [5]. These efforts contributed to the identification and development of two FDA-approved HDAC inhibitors, the most notable being a natural product, romidepsin [6].

**Figure 1:**
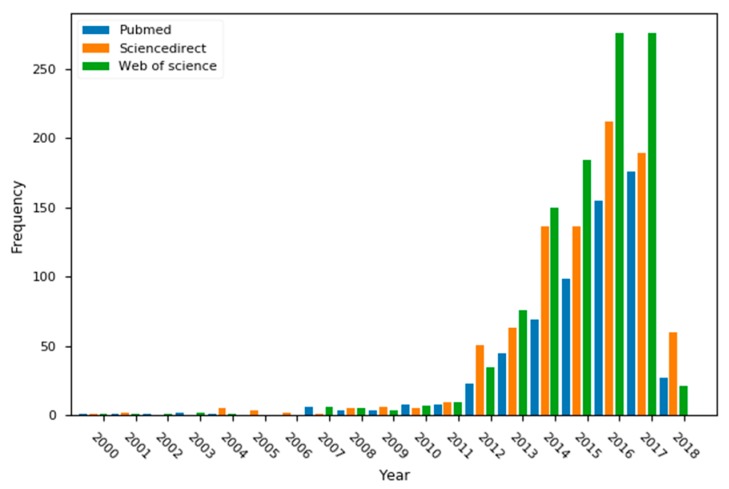
Frequency of the “bromodomain” keyword in three major search engines during the past 18 years.

**Figure 2:**
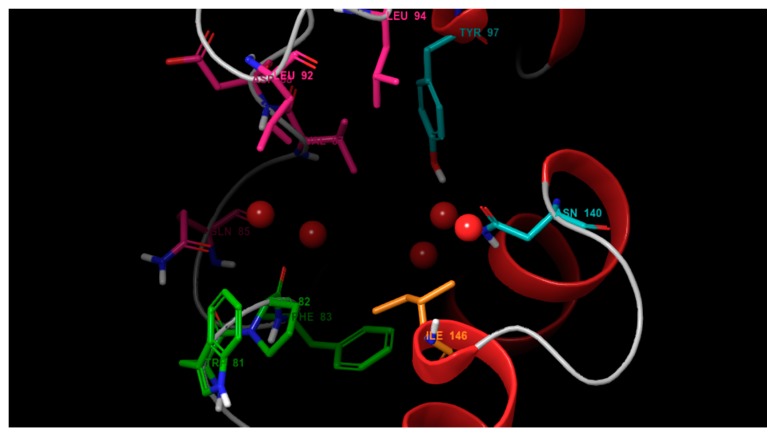
Binding pocket of bromodomain and extraterminal domain (BET) bromomains. The main structural features are the WPF shelf (green), ZA channel (pink), an Ac-pocket (cyan), and the gatekeeper (orange). Red spheres represent structural waters found with BET isoforms.

Readers are epi-enzymes whose function is to recognize certain modifications and their patterns on histones [7]. Therefore, reader enzymes are interesting molecular targets for a better understanding of epigenetics. Bromodomains are 120-residue proteins that were first discovered on the brahma (*brm*) gene of the *Drosophila* genus [8]. Later, it was confirmed as a common motif in most eukaryotic organisms. As of today, 62 isoforms were identified and are classified in eight families [9]. Family II, known as the bromodomain and extraterminal domain (BET), is extensively studied, as shown in Figure 1. This family includes bromodomain 2 (BRD2), BRD3, BRD4, and bromodomain testis-specific (BRDT) isoforms, each with their respective first and second domains (BD1 and BD2). Figure 2 shows the active site of bromodomains, which comprises three main hotspots: the WPF shelf, a region exclusive to BET bromodomains, a hydrophobic triad comprised by tryptophan, proline and phenylalanine (residues 80 to 83), and the ZA channel, located between the Z and A loops (residues 85 through 96), often seen as a frontier region with mixed contacts (mainly hydrophobic). The third hotspot is the Ac-binding pocket, responsible for reading histones and their ε-*N*-acetylated lysine residues (Kac). This hotspot is defined by a “tandem checkpoint” made by N140 and Y97 [10].

Additionally, evidence of structural water molecules was shown on a double bridge with ligands and Y97 [11]. Generally speaking, the role of water in binding is a dividing issue for drug design [12]. For example, structure-based design often ignores it or recognizes few instances of its importance [13]. Because of this, early approaches to ligand design followed a water-displacement strategy [14]. Nonetheless, a slow but steady paradigm shift came with increasing evidence of water-based stabilization in binding kinetics [15] and target selectivity [16]. One of the main problems in this approach is the increased difficulty of modeling of such phenomena, i.e., identifying “crucial waters” [17,18]. For bromodomains, recent studies showed that the network of structural waters in the ZA channel plays a significant role in binding [19,20] and this topic served as a case study for the development of novel methods in the field [21]. 

**Figure 3:**
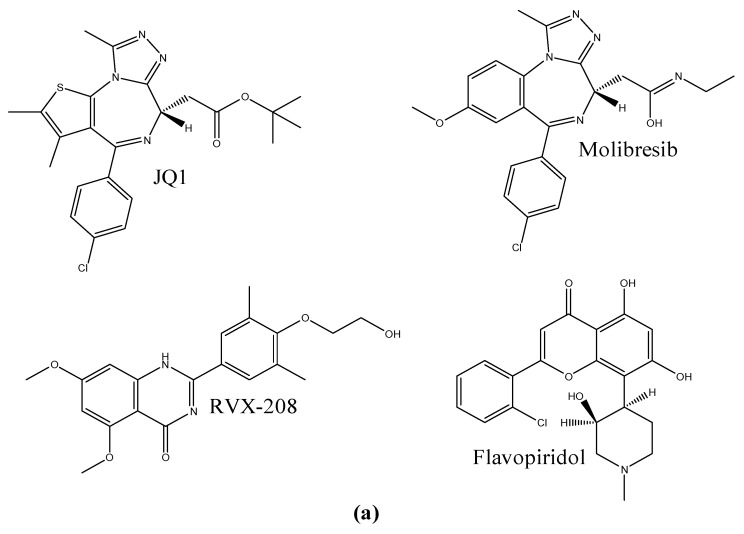

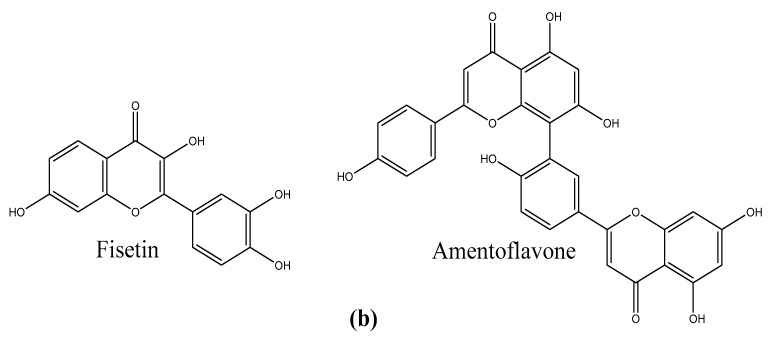
Chemical structures of (**a**) reference ligands for BET inhibition, and (**b**) flavonoids studied in this work.

Bromodomain inhibition is currently at an impasse [22], as chemotypes are not diverse enough to make more robust models and approaches toward their pharmacology. Hence, current efforts are focused on the synthesis and identification of plausible and novel inhibitors [23]. As part of this effort, quinazolones were proposed as novel inhibitors of BETs. An interesting property of these ligands is their selectivity toward BD2 [24]. Later, it was found that some kinase inhibitors can bind to bromodomains [25], e.g., flavopiridol. Figure 3a illustrates the quintessential BRD inhibitors. These results led to the hypothesis of flavonoids as putative modulators of bromodomains; nonetheless, this possibility was only explored in recent studies [26].

Flavonoids are one of the most well-known natural products, often regarded as major scaffolds in medicinal chemistry [27]. Flavonoids showed antioxidant [28], anti-inflammatory [29], and sedative [30] effects in various studies. Moreover, flavonoid scaffolds present the outstanding potential of being chemoprotective agents toward cancer [31]. Consequently, flavonoids are often seen as quintessential nutraceuticals; e.g., the average intake of flavonoids in the United States is around 1 g/day [32]. Finally, it was suggested that flavonoids may interact significantly with the epigenome; however, as of today, this is limited to writing and eraser epi-enzymes [33].

Fisetin, shown in Figure 3b, is a dietary flavonoid found in a broad array of vegetables such as strawberry, apple, grape, onion, and cucumber [34], and is considered a health-promoting compound [35]. Studies showed that fisetin is capable of blocking cell proliferation on many cancer lines [36]. One of the most interesting aspects of its pharmacology is its capacity to modulate nuclear factor kappa B (NF-κB) [37]. Fisetin is capable of doing this through the mitogen-activated protein kinase (MAPK) pathway and tumor necrosis factor (TNF)-blocking, downregulating pro-inflammatory genes [38]. Of note, recent studies showed the role of BRD4 in the recruitment of NF-κβ [39]. Thus, bromodomains were also studied for their role in chronic diseases like diabetes [40] and psoriasis [41]. Amentoflavone (Figure 3b) is a biflavonoid produced from two apigenin units. It is commonly found in *Ginko biloba*, *Hypericum perforatum*, *Biophytum sensitivum*, and *Nandina domestica* [42]. Like fisetin, amentoflavone was also identified as an NF-κβ modulator [43], thus giving rise to its capacity to reduce inflammation.

Computational methods are valuable approaches to solving chemical problems. Molecular docking, for example, allows the simulation of protein–ligand binding. Despite its simplifications and limitations, docking yields significant results used for binding-mode predictions [44]. Molecular dynamics is gaining increasing attention with regards to the elucidation of ligand binding and protein behavior [45]. 

Since amentoflavone and fisetin were identified as putative ligands of BRD4 in two independent studies [46,47], a comprehensive characterization of the putative binding profile of both flavonoids with BRD4 is presented herein. The binding profile was carried out with consensus docking and molecular dynamics. Based on the computational results, amentoflavone was experimentally tested for activity as BRD4 inhibitor, showing activity in the micromolar range. These results further support the activity of flavonoids as putative epi-modulators.

## 2. Materials and Methods 

### 2.1. Protein Preparation

An ensemble of 14 structures for the BET isoform, BRD4, was selected from the Protein Data Bank (PBD). Full details are presented in Table S1 of the Supplementary Materials. Selection criteria were based on their resolutions (<1.8 Å) and *R*-values (<0.25). Additional criteria were the structural similarity between the co-crystal ligand and the flavonoid scaffold, and the ability of the ligand to form hydrogen bonds with the binding pocket. All protein–ligand complexes were prepared with the Quickprep module of the MOE software [48]. Energy minimization was carried with the Amber 14: EHT force field (using Amber 14 forcefield [49] for protein parametrization and Extended Hückel Theory for ligands [50]). Complexes were visually inspected to ensure that key interactions were kept.

### 2.2. Molecular Docking

Docking was carried out using four programs: Autodock Vina [51], LeDock [52], MOE (v.2018.01), and PLANTS [53]. The rationale for selecting these programs was their performance and different scoring functions for consensus (vide infra). Protein inputs were kept from the preparation step and were validated with their respective native ligands. Details are provided in Table S2 of the Supplementary Materials. Amentoflavone and fisetin were parameterized with the Amber 14: EHT force field for the MOE software, and a charge reassignment was done with the LeDock, Vina, and PLANTS programs. The charge used for these programs was calculated with the MOPAC 2016 software [54] using PM6-D3H4X, as this correction was shown to enhance docking performance [55]. The docking poses were post-processed using protein–ligand interaction fingerprints (PLIF) as available in the MOE software. Docking poses were analyzed for clustering, based on the most common interactions found across the four programs.

### 2.3. Molecular Dynamics

Molecular dynamics simulations were carried out using Desmond [56] for both BRD4 (see Supplementary Materials, Figures S2, S3, and S6, and Table S12) and BRD4–ligand complexes. The complex used was the top ranked pose from the MOE software with consensus interactions. Complexes were then submitted to the System Builder utility in Maestro to assign a buffered 10 Å × 10 Å × 10 Å orthorhombic box using the transferable intermolecular potential with 3 points (TIP3P) water model and the OPLS_2005 force field. The system was neutralized, and a 0.15 M concentration of NaCl was added. Further details can be found in the Supplementary Materials, Figure S1. The production time for MD was set at 100 ns. The simulation was repeated three times. Electrostatics were computed using the Particle Mesh Ewald algorithm with a 9 Å cut-off, and constraints were enforced by the M-SHAKE algorithm [57]. Integration was done every 1.2 fs, with the recording interval set to 50 ps. The trajectories were then analyzed using the Simulation Interaction Diagram, Simulation Event Analysis, and Simulation Quality Analysis utilities in Maestro.

### 2.4. Experimental Testing of Amentoflavone

Amentoflavone was purchased from Sigma-Aldrich (St. Louis, MO, USA), and was tested for BRD4 tandem (BD1 + BD2) binding by means of AlphaScreen [58], using an H4 peptide (1–21) K5/8/12/16 Ac. Experimental work was performed by the Reaction Biology Corp., providing 2-mg samples to obtain duplicate dose-response curves beginning at a 100-μM concentration, following a three-fold dilution. The positive control for the test was the JQ-1 compound. The half maximal inhibitory concentration (IC_50_) values were obtained from the curves, and the Hill slope for amentoflavone was calculated. 

## 3. Results

### 3.1. Molecular Docking

**Figure 4:**
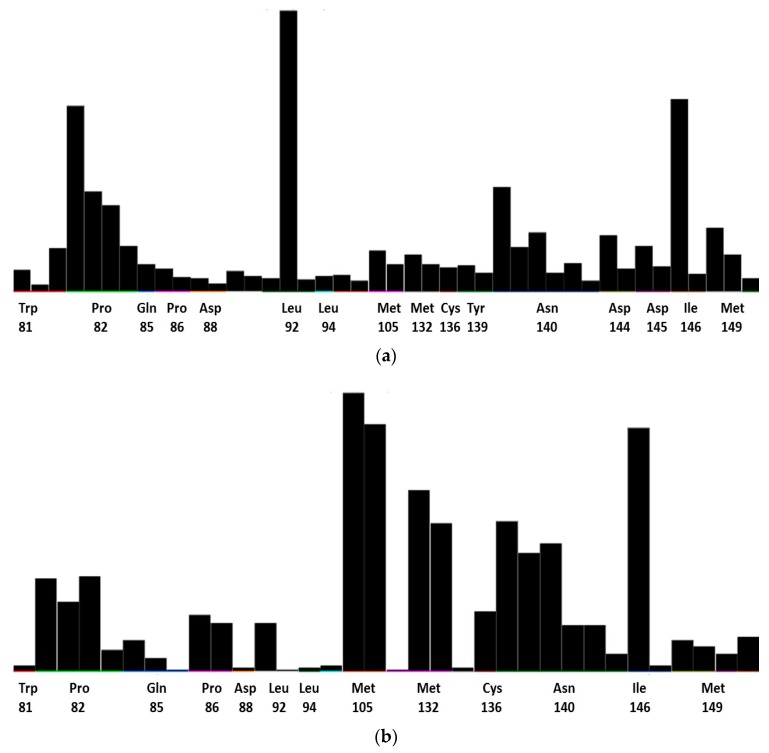
Consensus protein–ligand interaction fingerprint obtained from a consensus analysis of the docking with the Autodock Vina, LeDock, MOE, and PLANTS programs. (**a**) Amentoflavone; (**b**) Fisetin.

Table 1 summarizes the docking scores for amentoflavone and fisetin as computed with the four docking programs (the raw docking scores for each protein used are reported in the Supplementary Materials, Tables S4–S11). Figure 4 shows the consensus PLIF found for both compounds. 

### 3.2. Molecular Dynamics

**Figure 5:**
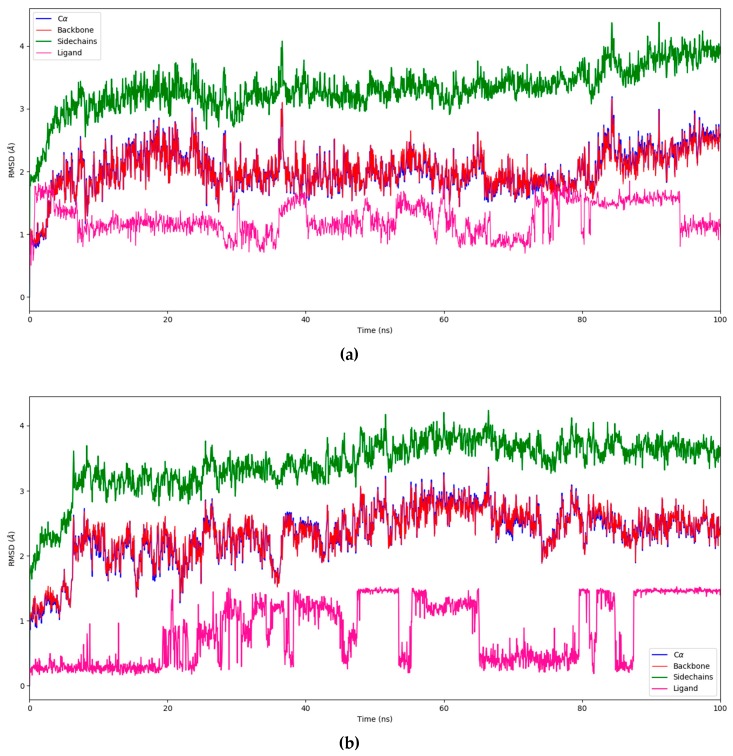
Root-mean-square deviation (RMSD) values for the protein backbone, alpha carbons, side chains, and ligand. (**a**) Amentoflavone; (**b**) Fisetin.

The overall quality of the MD simulations was measured with the corresponding utilities in Maestro. Energy, potential energy, temperature, pressure, and volume values were computed (results are shown in Figures S4 and S5, and Tables S13 and S14 in the Supplementary Materials). Once complex stability was assessed, the root-mean-square deviation (RMSD) values for backbone, Cα, side chains, and ligand were computed, as shown in Figure 5a,b. This measure shows the global deviation of atoms to a reference status (frame 0); usually, values below 5.0 Å can be considered as valid [59].

**Figure 6:**
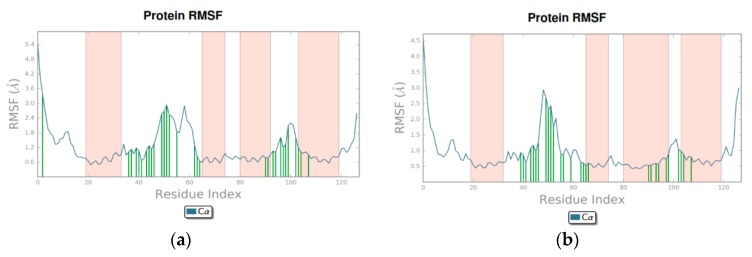
Root-mean-square fluctuation (RMSF) values based on alpha carbons; ligand contacts are presented in green, and protein helices in orange. (**a**) Amentoflavone; (**b**) Fisetin.

Root-mean-square fluctuation (RMSF) was also calculated, as shown in Figure 6a,b. These values show the general movement of each residue across the total simulation time. In this figure, the ligand contacts are shown as green lines matching the residue index, while the orange lines indicate protein secondary structures (helices, in this case). See Figure S6 in the Supplementary Materials for further details.

**Figure 7:**
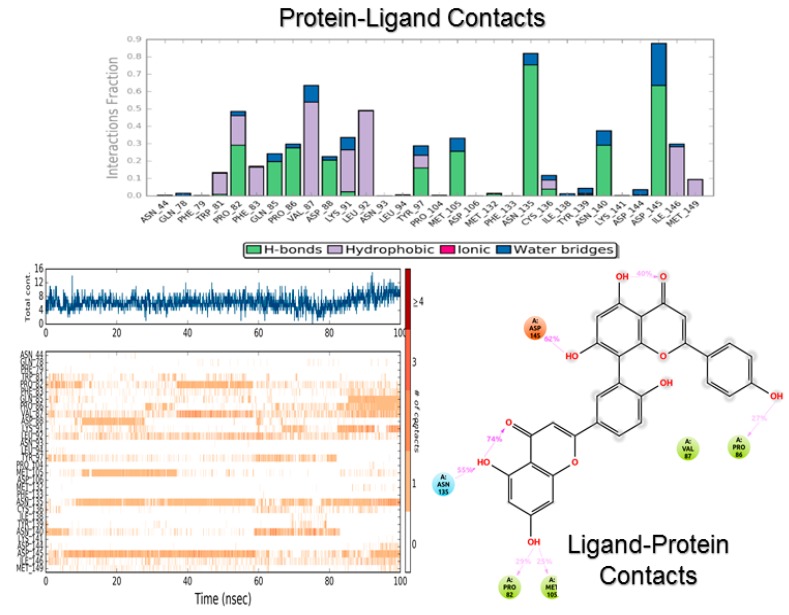
Protein–ligand contact analysis for amentoflavone during the molecular dynamics (MD) simulation.

**Figure 8:**
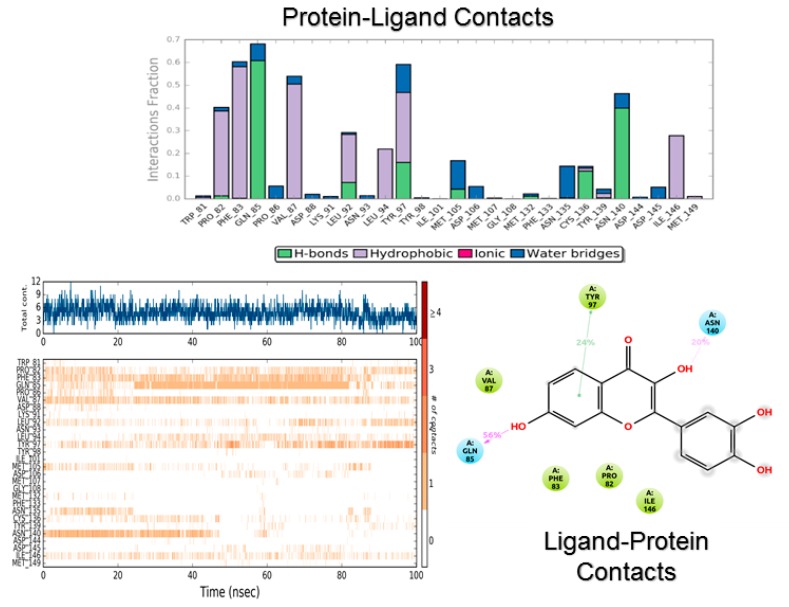
Protein–ligand contact analysis for fisetin during the MD simulation.

Figure 7 and Figure 8 show the protein–ligand contact analysis for the MD simulations. Protein–ligand contacts can be interpreted as “dynamic PLIFs”, showing the population of contacts during the simulation. Plots at the bottom of both figures represent the number of contacts and their density, i.e., a darker shade of orange indicates more than one contact in that frame. These plots also show the type of contact mapped to the structure of the ligand.

**Figure 9:**
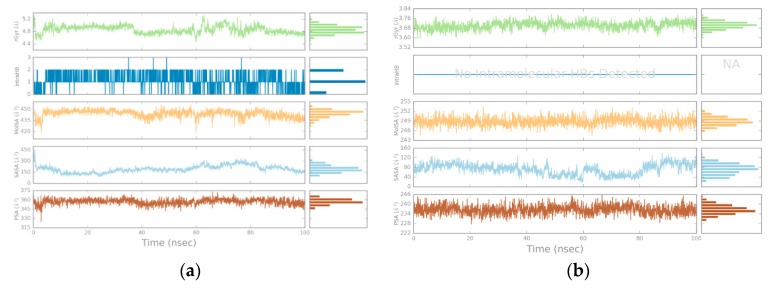
Ligand properties during the 100-ns simulations. (**a**) Amentoflavone; (**b**) Fisetin.

Figure 9a,b show other ligand properties during the MD simulations. These include the radius of gyration, intramolecular hydrogen bonding, van der Waals (VdW) surface area, solvent-accessible surface area, and polar surface area. Of note, if a ligand is not capable of intramolecular hydrogen bonding, this plot appears empty.

**Figure 10:**
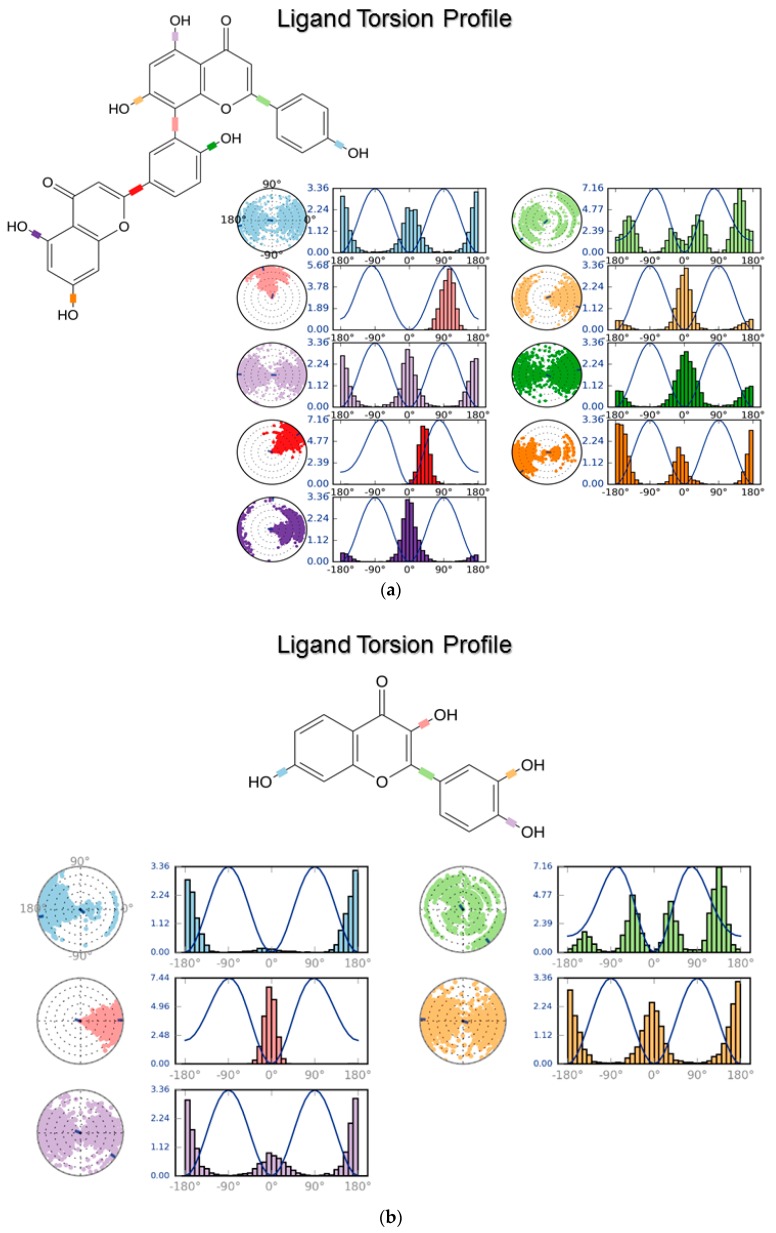
Torsional analysis of ligand conformations during the 100-ns simulations. (**a**) Amentoflavone; (**b**) Fisetin. The colors represent the different rotatable bonds of the ligands. Values on the Y-axis are in kcal/mol.

Figure 10a,b show the energy values for dihedral angles (line plot), which account for torsional analysis. The histogram shows the density of probability of that torsion, while the dial on the left shows the rotation of that bond during the simulation (the beginning is marked by the center). The plots in Figure 10 allow determining whether or not a given ligand undergoes torsional strain during binding.

### 3.3. Binding Assay of Amentoflavone

Table 2 summarizes the experimentally determined activity of amentoflavone.

## 4. Discussion

### 4.1. Molecular Docking

Bromodomain inhibitors may be classified into two broad categories: KAc-mimicking and non-mimicking, the former being the most prominent [60]. Flavonoids belong to this category as their carbonyl groups are their main anchor toward Asn140 and Tyr97 [46]. Nonetheless, as shown in Figure 4, these interactions are not as populated as might be expected. Certainly, this may seem negative, as the Ac-pocket is the main anchor for bromodomain inhibition. Nonetheless, the exclusion of molecules based solely on this criterion was questioned [61].

Because of this, an integral approach based on ensemble docking and consensus scoring was conducted, as a means of correctly assessing the probability of a given interaction. Ensemble docking is a common technique used to account for protein flexibility [62], and was applied successfully to several workflows [63]. Consensus scoring, on the other hand, significantly increases the rate of hit identification [64]. However, the rate of success has a strong dependence on the selected programs for consensus. Consequently, a naïve choice leads to an overestimation of weighted terms if similar parameters in scoring functions are used [65]. Hence, we selected the docking software based on searching-algorithm capabilities and scoring-function diversity. Briefly, the rationale for each selection is presented hereunder.

- Autodock VINA: It has a well-established performance against several protein families; additionally, its empirical scoring function has a significant correlation with experimental values [66]. Finally, its hybrid search algorithm optimized by local search allows a better sampling of the free-energy landscape [67].
- LeDock: Its search algorithm based on simulated annealing provides a significant clustering of poses. In addition, it was implemented successfully in virtual screening campaigns for BET bromodomains [52].
- MOE: Its docking algorithm allows for induced-fit search. Furthermore, its force-field-based scoring function (using AMBER parameters with generalized Born/volume integral (GB/VI) solvation) considers the solvation contributions to ligand binding [68].
- PLANTS: It provides a notable sampling of side-chain flexibility. Also, its searching algorithm (based on metaheuristics) and its empirical scoring function have a well-established performance [69].

Additionally, knowledge-based filtering was used to improve consensus results. This method takes advantage of PLIFs to identify trends in binding while selecting poses with “canonical” interactions [47]. Of note, the interactions of both flavonoids with Asn140 show a similar shift in MD simulations, which suggests a good sampling of our ensemble, and a notable performance of the protocol presented herein.

Based on the docking scores, both amentoflavone and fisetin are comparable to currently known inhibitors (see Supplementary Materials, Table S3). To the best of our knowledge, there are no studies or data showing a correlation of docking scores with experimental binding energies of bromodomain inhibitors. While such analysis goes beyond the scope of this work, we provided reference values obtained from the literature (see Supplementary Materials, Table S2). 

Notably, docking scores calculated with LeDock were lower when compared to the scores computed with other programs. Nevertheless, this same trend was observed for reference inhibitors. This result was mostly due to the scoring function, as it was shown that, while accurate for identifying correct binding poses, energy values assigned to them are often underestimated [70]. 

Additionally, the difference in score values for both compounds is significant. Roughly, these values suggest that amentoflavone could be three times more potent than fisetin. Arguably, this may be due to the bigger size of amentoflavone and the higher number of hydroxyl groups giving it more anchors toward BRD4. However, average scoring values rank them with a virtual IC_50_ of around 1–5 μM, based on scaffold similarity and reference values.

Amentoflavone showed mainly hydrophobic contacts with the WPF shelf (Pro81) and the ZA channel (Leu92). Fisetin on the other hand, showed more contacts with residues Met105 and Met132. Previous reports of docking indicate that flavonoids have a notable preference toward these residues [26]; in the case of fisetin, affinity for Cys136 was also observed [46]. This hints to flavonoids having a significant affinity toward different residues beyond the Ac-pocket, while their aromatic characteristics gives them selectivity for the WPF shelf.

Interestingly, the consensus PLIF (Figure 4) shows that amentoflavone made fewer contacts than fisetin. Moreover, the population of Asn140 bonding was significantly reduced for amentoflavone. In contrast, the MD simulations of both compounds showed a similar interaction profile, whereby the fraction is higher for amentoflavone (0.9 vs. 0.7). The Asn140-interaction fraction was similar for both flavonoids (around 0.4). Tyr97 on the other hand, made a stronger and more lasting interaction with fisetin by means of pi stacking and hydrogen bonding. This may be due to the size of amentoflavone and its orientation in the protein cavity, evidenced by the contacts with “non-canonical” residues. An example of this was Asp145, a contact with amentoflavone with a rather small population. However, this contact was identified as significant, as it provides ligand stabilization and water-network interactions [71]. 

### 4.2. Molecular Dynamics

As stated, MD simulations were conducted to contrast docking results, and to provide further insight into the binding mode of flavonoids. Based on the protein RMSD values, BRD4 remained stable enough during the simulation with both flavonoids. The ligand RMSD values, on the other hand, showed higher deviations at times. This could suggest that both ligands underwent conformational changes during the simulation, i.e., they had two binding modes. 

Therefore, torsional profiling plots assisted in this interpretation (Figure 10), as these provided the spatial and energetic distributions of bond torsions during the simulation, showing that both flavonoids were mostly strained in two main conformations. This could imply that fisetin changes its conformation more quickly than amentoflavone, due to a rotation of its catechol ring. However, this observation differs from a previous report [37], which suggested that fisetin keeps a restrained conformation when bound to BRD4. The main reason for this may be related to the use of different force fields (OPLS_2005 vs. OPLS3). On this matter, it is noteworthy that other ligand properties (Figure 9) showed similar trend values for fisetin to those in the previous study.

Amentoflavone, on the other hand, kept a restrained conformation of its shared phenol ring. This behavior can be related to atropisomerism features present on the biflavonoid. Based on these results, it can be hypothesized that amentoflavone activity on BETs is mediated by atropisomerism. Of course, stability studies of amentoflavone and its atropisomers are required to confirm this hypothesis. However, such techniques and focus were beyond the aims of this work. It suffices to say that, even though such phenomena may be common in bioflavonoids, their recognition in medicinal chemistry is often overlooked [72]. Moreover, the interest in atropisomerism is recently increasing [73]. Thus, we believe that knowledge of this feature could improve novel ligand designs, giving a paradigm shift mostly needed for these targets. 

The RMSF plots (Figure 6) also showed that the protein–ligand complexes remained consistently stable, and the main secondary structures were four α-helices, which confirms a correct sampling of the system. These plots also showed that the main contacts in both flavonoids were with the ZA channel, with high fluctuations of these residues during the simulation. Interestingly, when these protein–ligand contacts were analyzed, different interaction profiles arose for both flavonoids.

Amentoflavone clearly made more contacts with the ZA channel as the MD simulation went on. Also, its presence in the cavity made a significant impact on the secondary structure of the protein, increasing the helical portion of this region (Figure S6 in the Supporting Materials). As stated above, this may be related to the bigger size of the structure. However, based on the “contact mixture” this could also be related to the strained conformation of the molecule, allowing a more favorable angle toward hydrogen bonding and the hydrophobic interactions. 

Again, the contact with Asp145 is remarkable; in this case, it was the most populated contact in the MD simulations with amentoflavone. Also, the presence of water bridges with this residue proved significant, a feature recently observed by other groups [71]. Furthermore, this residue is present only in BRD4 BD1, providing specific contact with histone H3 via hydrogen bonding, an interaction not present with inhibitors such as JQ-1 [74]. This would suggest that amentoflavone can be selective for the first domain of BRD4. This is noteworthy, considering molecular similarity toward RVX-208 would suggest selectivity for BRD4 BD2.

### 4.3. Experimental Evaluation

Based on the results of molecular docking and dynamics, it was decided to acquire a sample, and to experimentally test amentoflavone as a BRD4 inhibitor. Fisetin was not considered for testing due to a previous report of quercetin showing an IC_50_ of 38 μM [58]. With this value as reference, our efforts focused on the biflavonoid scaffold. It is very positive that amentoflavone showed significant binding for BRD4, with an IC_50_ in the micromolar range. Indeed, it was more potent when compared to a flavonoid monomer (38 vs. 30 μM). Additionally, its Hill slope value could indicate that amentoflavone is indeed selective for one domain of BRD4. However, more testing is required, i.e., binding assays for separate domains of BRD4. 

Furthermore, this experimental confirmation provides further evidence of flavonoids as general chemoprotective agents. One of the main concerns about the use of flavonoids as nutraceutics is their putative toxicity, as fisetin and other flavonoid monomers inhibit DNA topoisomerases [75] and actin polymerization [36]. Biflavonoids, on the other hand, do not present this feature; however, they were reported as potentially mutagenic [76]. Nonetheless, such negative effects are only present at concentrations between 100 and 250 μM [77,78]. As such, based on the IC_50_ values of both quercetin and amentoflavone, flavonoids have significant potential as epi-nutraceutics.

Summarizing, the work presented here serves as a remarkable proof of concept for both the flavonoids as epi-modulators and the computational methods used herein. Putting the results together, amentoflavone showed the characteristic contacts previously reported for flavonoids, i.e., strong contacts with the ZA region, in addition to novel predicted interactions with Asp145 and the water network. Biological tests supported the hypothesis of binding and the plausible selectivity.

Despite the fact that flavonoids have small room for optimization and break Lipinski’s rule of five, their true potential is as chemoprotective agents. As previously mentioned, this finding further advances the field of nutriepigenomics. Moreover, it is remarkable that these natural products provide pharmacophoric templates for novel inhibitors of an epigenetic target. 

## 5. Conclusions

Amentoflavone is a natural product with several associated biological effects. Its ability to block NF-κβ is the key for its anti-inflammatory potential. BETs were identified as NF-κβ promoters, with JQ-1 being highly effective in psoriasis models. Previously, a similar effect was reported for amentoflavone. Based on these results and other reports, we conducted a binding characterization of this ligand, and compared it to fisetin, another flavonoid with reports of putative activity. We presented a consensus docking methodology which allowed binding characterization and hit selection. Certainly, such an approach is impractical for large virtual-screening campaigns. However, based on the performance and results presented, it provides a powerful tool for pose selection, as supported by the MD results. 

The simulations conducted herein indicated that amentoflavone can make numerous contacts in the ZA channel, as previously described for flavonoids and kinase inhibitors. It was also determined that amentoflavone can potentially make contacts with “non-canonical” residues for BET inhibition, e.g., Met105, Asn135, Cys136, and Asp145. Most of these contacts were not observed with fisetin (except for Cys136). Based on the analysis of torsional values, it is plausible that this behavior was due to the atropisomerism present in the molecule. As a first step toward testing this hypothesis, the in vitro inhibition of BRD4 was evaluated. The experimental evaluation showed that amentoflavone was indeed active in the micromolar range, with plausible selectivity against one domain in the BRD4 tandem.

Perspectives of this work include the experimental testing of fisetin and the contrast of its result with molecular modeling predictions. Additionally, for amentoflavone, specific tests for BD1 and BD2 are required to confirm its selectivity. Finally, we consider that these results, while preliminary, offer a new paradigm for inhibitor design, as well as characteristics for novel modulation of BETs. 

## Figures and Tables

**Table 1:** Summary statistics of docking scores for the programs used.

Molecule	Summary Stats *	Autodock VINA (kcal/mol)	LeDock (kcal/mol)	MOE (kcal/mol)	PLANTS
Amentoflavone	Min	−10.5	−7.9	−9.0	−102.1
	1Q	−9.5	−7.3	−7.9	−89.4
	Avg	−9.2	−7.0	−7.6	−86.9
	3Q	−9.0	−6.8	−7.2	−84.0
	Max	−8.2	−6.3	−6.4	−77.4
	SD	0.46	0.34	0.54	4.6
Fisetin	Min	−8.6	−6.0	−7.4	−79.6
	1Q	−8.2	−5.6	−6.5	−73.2
	Avg	−7.9	−5.4	−6.2	−71.0
	3Q	−7.7	−5.3	−6.0	−68.9
	Max	−7.1	−4.7	−5.6	−65.0
	SD	0.31	0.24	0.39	2.94

* Min: Minimum; 1Q: First quartile; Avg: Average; 3Q: Third quartile; Max: Maximum; and SD: Standard deviation values.

**Table 2:** Values for the half maximal inhibitory concentration (IC_50_) and the Hill slope of amentoflavone, as obtained using AlphaScreen ^†^ against the bromodomain 4 (BRD4) tandem.

	DATA 1	DATA 2
IC_50_ (μM)	36.1	30.4
Hill slope	−2.5	−1.9

^†^ As mentioned in the Methods section, experimental characterization was performed with the Reaction Biology application using the AlphaScreen assay.

## Supplementary Materials

The following are available online at http://www.mdpi.com/2218-273X/8/3/61/s1. Figure S1: Relaxation protocol and MD workflow used in this work, Table S1: BRD4 PDBIDs used for cross-docking studies Figure S2: Simulation Quality parameters for the BRD4 protein for 100 ns, Table S2: IC_50_ and experimental binding energy values and of reference ligands for BRD4 inhibition as reported on the literature, Figure S3: RMSD values for BRD4 protein for 100ns, Table S3: Scoring values for reference ligands as obtained by the docking software used herein, Figure S4: Simulation Quality parameters for the BRD4 protein with fisetine for 100 ns, Table S4: Scoring values obtained with LeDock for amentoflavone per protein-ligand complex of the ensemble, Figure S5: Simulation Quality parameters for the BRD4 protein with amentoflavone for 100 ns, Table S5: Scoring values obtained with MOE for amentoflavone per protein-ligand complex of the ensemble, Figure S6: Secondary structure of BRD4 as observed for 100 ns. A) BRD4 without ligand. B) amentoflavone. C) fisetin., Table S6: Scoring values obtained with PLANTS for amentoflavone per protein-ligand complex of the ensemble, Table S7: Scoring values obtained with Vina for amentoflavone per protein-ligand complex of the ensemble, Table S8: Scoring values obtained with LeDock for fisetin per protein-ligand complex of the ensemble, Table S9: Scoring values obtained with MOE for fisetin per protein-ligand complex of the ensemble, Table S10: Scoring values obtained with PLANTS for fisetin per protein-ligand complex of the ensemble, Table S11: Scoring values obtained with Vina for fisetin per protein-ligand complex of the ensemble, Table S12: Summary values for quality measures for the BRD4 protein, Table S13: Summary values for quality measures BRD4 protein with fisetine, Table S14: Summary values for quality measures BRD4 protein with amentoflavone.
