# Supplementary material for: Flavonoids as Putative Epi-Modulators: Insight into Their Binding Mode with BRD4 Bromodomains Using Molecular Docking and Dynamics
Source: Biomolecules. 2018 Jul 23;8(3):61. doi: 10.3390/biom8030061 (PMC6164663; doi:10.3390/biom8030061)

Supplementary Information

Flavonoids as putative epi-modulators: Insights into their binding mode with BRD4 bromodomain using molecular docking and dynamics.

Fernando D. Prieto-Martínez*, José L. Medina-Franco*

Facultad de Química, Departamento de Farmacia, Universidad Nacional Autónoma de México, Avenida Universidad 3000, Mexico City 04510, Mexico

*Contact authors: Phone: +5255-5622-3899.

E-mails: fprieto@comunidad.unam.mx (Prieto-Martínez), medinajl@unam.com.mx (Medina-Franco).

Contents

Table S1. BRD4 PDBIDs used for cross-docking studies

Table S2. IC_{50} and experimental binding energy values and of reference ligands for BRD4 inhibition as reported on the literature.

Table S3. Scoring values for reference ligands as obtained by the docking software used herein.

Table S4. Scoring values obtained with LeDock for amentoflavone per protein-ligand complex of the ensemble

Table S5. Scoring values obtained with MOE for amentoflavone per protein-ligand complex of the ensemble

Table S6. Scoring values obtained with PLANTS for amentoflavone per protein-ligand complex of the ensemble

Table S7. Scoring values obtained with Vina for amentoflavone per protein-ligand complex of the ensemble

Table S8. Scoring values obtained with LeDock for fisetin per protein-ligand complex of the ensemble

Table S9. Scoring values obtained with MOE for fisetin per protein-ligand complex of the ensemble

Table S10. Scoring values obtained with PLANTS for fisetin per protein-ligand complex of the ensemble

Table S11. Scoring values obtained with Vina for fisetin per protein-ligand complex of the ensemble

Table S12. Summary values for quality measures for the BRD4 protein

Table S13. Summary values for quality measures BRD4 protein with fisetine

Table S14. Summary values for quality measures BRD4 protein with amentoflavone

Figure S1. Relaxation protocol and MD workflow used in this work.

Figure S2. Simulation Quality parameters for the BRD4 protein for 100 ns

Figure S3. RMSD values for BRD4 protein for 100ns

Figure S4. Simulation Quality parameters for the BRD4 protein with fisetine for 100 ns

Figure S5. Simulation Quality parameters for the BRD4 protein with amentoflavone for 100 ns

Figure S6. Secondary structure of BRD4 as observed for 100 ns. A) BRD4 without ligand. B) amentoflavone. C) fisetin

Table S1. BRD4 PDBIDs used for cross-docking studies

ID	*Resolution(Å)*	*R-factor*
3MXF	1.6	0.184
3P5O	1.6	0.185
3U5L	1.39	0.142
4LYW	1.95	0.235
4MR4	1.66	0.194
4NUD	1.2	0.171
4XYA	2.05	0.207
4YH3	1.6	0.199
5KJ0	1.51	0.17
5M3A	1.65	0.214
5TI2	1.65	0.181
5TI3	1.703	0.194
5U28	1.798	0.223
5VOM	1.67	0.218

Table S2. IC_{50} and experimental binding energy values and of reference ligands for BRD4 inhibition as reported on the literature.

Ligand	IC_{50} (nM)	ΔG (kcal/mol)
**JQ-1**	**60**	**-9.64**
**Molibresib**	**40**	**N/A**
**BZT-7**	**640**	**-8.16**
**XD14**	**N/A**	**-9.0**
**RVX-208**	**645**	**-7.84**
**MS436**	**~4000**	**N/A**
**43S**	**N/A**	**-7.73**
**CHEMBL3589468**	**9000**	**N/A**
**6TB**	**130**	**N/A**
**7E7**	**2000**	**N/A**
**ZINC5329468**	**240**	**N/A**
**7CG**	**44**	**N/A**
**SF2523**	**240**	**N/A**
**9GY**	**790**	**N/A**

Table S3. Scoring values for reference ligands as obtained by the docking software used herein.

MOLECULE	SUMMARY STATS	AUTODOCK VINA	LEDOCK	MOE	PLANTS
**JQ-1**	Min:	-9.0 kcal/mol	-5.9 kcal/mol	-8.5 kcal/mol	-102.1
	1st Quart.:	-7.6 kcal/mol	-5.5 kcal/mol	-7.4 kcal/mol	-80.7
	Avg:	-7.3 kcal/mol	-5.0 kcal/mol	-6.9 kcal/mol	-74.0
	3rd Quart.:	-6.8 kcal/mol	-4.5 kcal/mol	-6.4 kcal/mol	-68.0
	Max:	-5.8 kcal/mol	-4.2 kcal/mol	-5.3 kcal/mol	-50.4
	SD:	0.7 kcal/mol	0.6 kcal/mol	0.7 kcal/mol	11.8

**MOLIBRESIB**	Min:	-9.3 kcal/mol	-6.3 kcal/mol	-8.3 kcal/mol	-102.8
	1st Quart.:	-8.2 kcal/mol	-6.2 kcal/mol	-7.4 kcal/mol	-83.2
	Avg:	-7.6 kcal/mol	-5.7 kcal/mol	-6.9 kcal/mol	-75.6
	3rd Quart.:	-7.1 kcal/mol	-5.3 kcal/mol	-6.4 kcal/mol	-67.8
	Max:	-6.5 kcal/mol	-4.6 kcal/mol	-5.3 kcal/mol	-62.0
	SD:	0.7 kcal/mol	0.5 kcal/mol	0.7 kcal/mol	10.3
**BZT-7**	Min:	-9.3 kcal/mol	-5.5 kcal/mol	-7.2 kcal/mol	-92.8
	1st Quart.:	-8.0 kcal/mol	-4.9 kcal/mol	-6.0 kcal/mol	-77.6
	Avg:	-7.7 kcal/mol	-4.5 kcal/mol	-5.6 kcal/mol	-72.2
	3rd Quart.:	-7.2 kcal/mol	-4.2 kcal/mol	-5.1 kcal/mol	-65.7
	Max:	-6.3 kcal/mol	-3.7 kcal/mol	-4.3 kcal/mol	-50.6
	SD:	0.7 kcal/mol	0.5 kcal/mol	0.6 kcal/mol	12.0
**XD14**	Min:	-7.7 kcal/mol	-7.0 kcal/mol	-7.8 kcal/mol	-96.7
	1st Quart.:	-6.9 kcal/mol	-6.4 kcal/mol	-6.8 kcal/mol	-80.2
	Avg:	-6.6 kcal/mol	-6.0 kcal/mol	-6.5 kcal/mol	-76.1
	3rd Quart.:	-6.3 kcal/mol	-5.8 kcal/mol	-6.2 kcal/mol	-71.7
	Max:	-6.0 kcal/mol	-4.4 kcal/mol	-5.8 kcal/mol	-64.5
	SD:	0.4 kcal/mol	0.4 kcal/mol	0.4 kcal/mol	7.0
**RVX-208**	Min:	-7.7 kcal/mol	-6.5 kcal/mol	-7.2 kcal/mol	-69.4
	1st Quart.:	-7.0 kcal/mol	-6.0 kcal/mol	-6.5 kcal/mol	-61.8
	Avg:	-6.8 kcal/mol	-5.6 kcal/mol	-6.2 kcal/mol	-57.4
	3rd Quart.:	-6.7 kcal/mol	-5.3 kcal/mol	-5.9 kcal/mol	-52.2
	Max:	-6.4 kcal/mol	-4.5 kcal/mol	-5.6 kcal/mol	-46.4
	SD:	0.2 kcal/mol	0.4 kcal/mol	0.4 kcal/mol	6.2
**MS436**	Min:	-9.0 kcal/mol	-6.6 kcal/mol	-6.8 kcal/mol	-84.4
	1st Quart.:	-8.2 kcal/mol	-6.1 kcal/mol	-6.2 kcal/mol	-79.7
	Avg:	-8.0 kcal/mol	-6.0 kcal/mol	-5.9 kcal/mol	-75.9
	3rd Quart.:	-7.7 kcal/mol	-5.8 kcal/mol	-5.6 kcal/mol	-73.2
	Max:	-7.3 kcal/mol	-5.3 kcal/mol	-5.1 kcal/mol	-64.9
	SD:	0.3 kcal/mol	0.2 kcal/mol	0.4 kcal/mol	5.0
**43S**	Min:	-8.8 kcal/mol	-6.4 kcal/mol	-7.0 kcal/mol	-74.5
	1st Quart.:	-8.1 kcal/mol	-6.1 kcal/mol	-6.0 kcal/mol	-71.0
	Avg:	-7.7 kcal/mol	-5.8 kcal/mol	-5.7 kcal/mol	-68.3
	3rd Quart.:	-7.5 kcal/mol	-5.6 kcal/mol	-5.4 kcal/mol	-65.4
	Max:	-6.7 kcal/mol	-5.1 kcal/mol	-4.7 kcal/mol	-62.9
	SD:	0.4 kcal/mol	0.4 kcal/mol	0.4 kcal/mol	3.3
**CHEMBL3589468**	Min:	-9.6 kcal/mol	-5.3 kcal/mol	-7.3 kcal/mol	-94.2
	1st Quart.:	-8.2 kcal/mol	-5.0 kcal/mol	-6.3 kcal/mol	-74.4
	Avg:	-7.9 kcal/mol	-4.7 kcal/mol	-6.2 kcal/mol	-67.8
	3rd Quart.:	-7.6 kcal/mol	-4.4 kcal/mol	-5.9 kcal/mol	-61.2
	Max:	-6.8 kcal/mol	-4.2 kcal/mol	-5.6 kcal/mol	-44.9
	SD:	0.5 kcal/mol	0.3 kcal/mol	0.3 kcal/mol	11.0
**6TB**	Min:	-8.3 kcal/mol	-6.3 kcal/mol	-9.4 kcal/mol	-63.2
	1st Quart.:	-7.0 kcal/mol	-5.7 kcal/mol	-7.4 kcal/mol	-51.3
	Avg:	-6.8 kcal/mol	-5.4 kcal/mol	-7.1 kcal/mol	-46.3
	3rd Quart.:	-6.6 kcal/mol	-5.0 kcal/mol	-6.7 kcal/mol	-41.0
	Max:	-6.1 kcal/mol	-4.4 kcal/mol	-6.1 kcal/mol	-34.8
	SD:	0.3 kcal/mol	0.5 kcal/mol	0.6 kcal/mol	6.4

**7E7**	Min:	-8.9 kcal/mol	-6.3 kcal/mol	-7.0 kcal/mol	-69.9
	1st Quart.:	-8.0 kcal/mol	-5.8 kcal/mol	-6.3 kcal/mol	-61.9
	Avg:	-7.7 kcal/mol	-5.7 kcal/mol	-6.1 kcal/mol	-60.0
	3rd Quart.:	-7.4 kcal/mol	-5.4 kcal/mol	-5.8 kcal/mol	-57.3
	Max:	-6.8 kcal/mol	-5.2 kcal/mol	-5.5 kcal/mol	-52.5
	SD:	0.4 kcal/mol	0.3 kcal/mol	0.3 kcal/mol	4.2
**ZINC5329468**	Min:	-8.6 kcal/mol	-5.5 kcal/mol	-6.8 kcal/mol	-80.0
	1st Quart.:	-8.0 kcal/mol	-4.9 kcal/mol	-6.2 kcal/mol	-75.9
	Avg:	-7.7 kcal/mol	-4.7 kcal/mol	-6.0 kcal/mol	-73.7
	3rd Quart.:	-7.4 kcal/mol	-4.4 kcal/mol	-5.8 kcal/mol	-71.6
	Max:	-6.9 kcal/mol	-4.0 kcal/mol	-5.4 kcal/mol	-65.8
	SD:	0.4 kcal/mol	0.3 kcal/mol	0.3 kcal/mol	3.6
**7CG**	Min:	-7.9 kcal/mol	-6.0 kcal/mol	-7.0 kcal/mol	-80.9
	1st Quart.:	-7.4 kcal/mol	-5.6 kcal/mol	-6.1 kcal/mol	-75.2
	Avg:	-7.2 kcal/mol	-5.4 kcal/mol	-6.0 kcal/mol	-73.0
	3rd Quart.:	-7.0 kcal/mol	-5.3 kcal/mol	-5.7 kcal/mol	-70.6
	Max:	-6.5 kcal/mol	-4.9 kcal/mol	-5.4 kcal/mol	-65.9
	SD:	0.2 kcal/mol	0.3 kcal/mol	0.3 kcal/mol	3.7
**SF2523**	Min:	-8.8 kcal/mol	-5.7 kcal/mol	-7.3 kcal/mol	-75.8
	1st Quart.:	-8.1 kcal/mol	-5.3 kcal/mol	-6.4 kcal/mol	-69.2
	Avg:	-7.7 kcal/mol	-4.9 kcal/mol	-6.2 kcal/mol	-66.6
	3rd Quart.:	-7.4 kcal/mol	-4.5 kcal/mol	-6.0 kcal/mol	-63.0
	Max:	-6.5 kcal/mol	-4.1 kcal/mol	-5.3 kcal/mol	-53.9
	SD:	0.4 kcal/mol	0.5 kcal/mol	0.3 kcal/mol	4.6
**9GY**	Min:	-10.3 kcal/mol	-5.7 kcal/mol	-8.2 kcal/mol	-105.5
	1st Quart.:	-9.2 kcal/mol	-5.2 kcal/mol	-7.2 kcal/mol	-96.2
	Avg:	-8.8 kcal/mol	-5.0 kcal/mol	-6.9 kcal/mol	-85.6
	3rd Quart.:	-8.3 kcal/mol	-4.9 kcal/mol	-6.5 kcal/mol	-73.1
	Max:	-7.7 kcal/mol	-4.5 kcal/mol	-5.8 kcal/mol	-54.4
	SD:	0.6 kcal/mol	0.2 kcal/mol	0.5 kcal/mol	14.2

Table S4. Scoring values obtained with LeDock for amentoflavone per protein-ligand complex of the ensemble

3MXF	3P5O	3U5L	4LYW	4MR4	4NUD	4XYA	4YH3	5KJ0	5M3A	5TI2	5TI3	5U28	5VOM
-7.4	-7.7	-7.7	-7.5	-7.4	-7.3	-7.4	-7.0	-7.4	-7.7	-7.7	-7.9	-7.4	-7.3
-7.4	-7.7	-7.4	-7.3	-7.4	-7.3	-7.2	-6.8	-7.3	-7.7	-7.5	-7.4	-7.3	-7.0
-7.2	-7.4	-7.3	-7.3	-7.3	-7.2	-7.0	-6.8	-7.2	-7.5	-7.5	-7.4	-7.1	-6.8
-7.0	-7.2	-6.8	-7.2	-7.1	-7.2	-6.9	-6.8	-7.2	-7.4	-7.4	-7.3	-7.1	-6.8
-7.0	-7.1	-6.8	-7.0	-7.0	-7.2	-6.8	-6.7	-7.1	-7.4	-7.4	-7.1	-7.0	-6.5
-6.9	-7.1	-6.7	-6.9	-7.0	-7.2	-6.8	-6.7	-7.0	-7.3	-7.3	-6.9	-6.8	-6.4
-6.6	-6.9	-6.7	-6.9	-6.9	-7.0	-6.5	-6.6	-7.0	-7.2	-7.3	-6.9	-6.7	-6.4
-6.5	-6.9	-6.6	-6.8	-6.8	-7.0	-6.5	-6.5	-6.9	-7.2	-7.2	-6.8	-6.6	-6.4
-6.4	-6.9	-6.6	-6.7	-6.8	-7.0	-6.5	-6.5	-6.8	-7.1	-7.0	-6.8	-6.5	-6.3

Table S5. Scoring values obtained with MOE for amentoflavone per protein-ligand complex of the ensemble

3MXF	3P5O	3U5L	4LYW	4MR4	4NUD	4XYA	4YH3	5KJ0	5M3A	5TI2	5TI3	5U28	5VOM
-8.8	-8.4	-8.0	-8.5	-7.8	-8.4	-9.0	-8.3	-8.2	-8.0	-7.8	-7.8	-8.8	-8.6
-8.7	-8.0	-8.0	-7.6	-7.5	-8.4	-8.3	-8.2	-8.2	-7.9	-7.5	-7.8	-8.4	-8.4
-8.0	-7.6	-7.3	-7.5	-7.3	-8.3	-8.2	-7.8	-8.1	-7.9	-7.4	-7.4	-8.3	-8.2
-7.9	-7.4	-7.3	-7.3	-7.3	-7.6	-8.0	-7.8	-8.1	-7.5	-7.4	-7.3	-8.2	-8.2
-7.8	-7.4	-7.2	-7.2	-7.3	-7.4	-7.8	-7.8	-8.1	-7.4	-7.2	-7.1	-8.2	-8.0
-7.7	-7.4	-7.2	-7.0	-7.0	-7.0	-7.5	-7.8	-7.8	-7.3	-7.0	-7.1	-7.9	-7.7
-7.4	-7.2	-7.1	-7.0	-6.8	-6.9	-7.4	-7.7	-7.8	-7.2	-7.0	-7.0	-7.9	-7.7
-7.4	-7.2	-7.0	-6.8	-6.6	-6.7	-7.4	-7.5	-7.3	-7.0	-7.0	-7.0	-7.8	-7.7
-7.2	-7.0	-6.8	-6.5	-6.4	-6.6	-7.1	-7.4	-7.3	-7.0	-6.7	-6.7	-7.4	-7.5

Table S6. Scoring values obtained with PLANTS for amentoflavone per protein-ligand complex of the ensemble

3MXF	3P5O	3U5L	4LYW	4MR4	4NUD	4XYA	4YH3	5KJ0	5M3A	5TI2	5TI3	5U28	5VOM
-94.4	-94.9	-93.5	-98.5	-92.4	-91.6	-89.4	-92.8	-93.8	-102.1	-92.8	-98.0	-93.3	-84.5
-89.4	-94.3	-89.6	-96.7	-91.4	-91.0	-87.3	-91.1	-93.4	-101.1	-91.2	-91.5	-89.1	-82.8
-88.8	-90.8	-86.4	-89.3	-89.0	-90.5	-85.5	-91.0	-90.6	-96.6	-87.7	-90.0	-88.9	-80.8
-87.4	-86.2	-85.1	-88.4	-88.0	-89.5	-84.7	-90.8	-87.4	-95.1	-87.4	-85.3	-88.7	-79.7
-87.2	-85.4	-84.2	-86.8	-86.3	-88.4	-84.5	-90.2	-86.1	-94.3	-85.8	-85.1	-87.3	-78.0
-86.8	-85.0	-83.6	-86.7	-86.0	-87.6	-84.4	-89.2	-84.0	-90.7	-82.6	-85.0	-87.0	-78.0
-86.8	-84.6	-83.4	-86.6	-85.9	-85.8	-84.2	-87.5	-83.8	-90.6	-81.8	-85.0	-85.7	-77.9
-84.9	-84.5	-82.7	-86.6	-84.6	-85.2	-83.2	-87.4	-83.7	-88.3	-81.8	-84.0	-85.6	-77.8
-83.3	-82.7	-82.6	-84.4	-84.2	-84.0	-83.0	-84.4	-83.3	-86.3	-81.0	-83.8	-85.2	-77.6

Table S7. Scoring values obtained with Vina for amentoflavone per protein-ligand complex of the ensemble

3MXF	3P5O	3U5L	4LYW	4MR4	4NUD	4XYA	4YH3	5KJ0	5M3A	5TI2	5TI3	5U28	5VOM
-9.9	-10.2	-9.7	-10.1	-9.5	-10.0	-9.6	-9.6	-9.8	-10.0	-10.5	-10.5	-9.5	-9.5
-9.5	-10.0	-9.6	-10.0	-9.5	-10.0	-9.5	-9.4	-9.5	-9.9	-9.5	-10.0	-9.4	-9.0
-9.5	-9.4	-9.4	-9.9	-9.5	-10.0	-9.4	-9.3	-9.3	-9.9	-9.4	-9.8	-9.3	-8.9
-9.4	-9.3	-9.4	-9.6	-9.4	-9.9	-9.2	-9.3	-9.2	-9.8	-9.4	-9.4	-9.2	-8.4
-9.4	-9.2	-9.1	-9.5	-9.3	-9.5	-9.2	-9.3	-9.2	-9.5	-9.1	-9.1	-9.0	-8.4
-9.4	-9.0	-9.0	-9.5	-9.2	-9.2	-9.0	-9.2	-8.9	-9.5	-9.1	-9.0	-9.0	-8.3
-9.3	-9.0	-9.0	-9.3	-9.1	-9.1	-8.8	-9.0	-8.6	-9.5	-9.0	-9.0	-8.7	-8.2
-9.3	-8.9	-8.8	-9.3	-9.0	-8.7	-8.7	-8.9	-8.4	-9.5	-9.0	-9.0	-8.5	-8.2
-9.2	-8.7	-8.8	-9.2	-8.9	-8.7	-8.7	-8.9	-8.3	-9.4	-8.8	-8.9	-8.5	-8.2

Table S8. Scoring values obtained with LeDock for fisetin per protein-ligand complex of the ensemble

3MXF	3P5O	3U5L	4LYW	4MR4	4NUD	4XYA	4YH3	5KJ0	5M3A	5TI2	5TI3	5U28	5VOM
-5.8	-5.6	-5.5	-5.7	-5.7	-5.8	-5.7	-5.3	-5.6	-6.0	-6.0	-6.0	-5.5	-5.7
-5.8	-5.6	-5.5	-5.6	-5.7	-5.8	-5.7	-5.2	-5.6	-5.9	-6.0	-6.0	-5.5	-5.7
-5.6	-5.5	-5.5	-5.6	-5.6	-5.7	-5.6	-5.2	-5.5	-5.9	-5.8	-5.8	-5.4	-5.7
-5.6	-5.4	-5.4	-5.6	-5.3	-5.6	-5.6	-5.1	-5.5	-5.6	-5.5	-5.5	-5.3	-5.6
-5.5	-5.3	-5.3	-5.5	-5.3	-5.5	-5.5	-5.1	-5.4	-5.5	-5.4	-5.4	-5.2	-5.6
-5.4	-5.3	-5.3	-5.5	-5.3	-5.5	-5.5	-5.1	-5.4	-5.5	-5.4	-5.4	-5.2	-5.4
-5.4	-5.3	-5.3	-5.5	-5.2	-5.2	-5.5	-5.0	-5.4	-5.5	-5.2	-5.3	-5.0	-5.4
-5.4	-5.2	-5.3	-5.5	-5.2	-5.1	-5.4	-4.8	-5.4	-5.4	-5.2	-5.2	-5.0	-5.3
-5.3	-5.2	-5.3	-5.4	-5.1	-5.0	-5.4	-4.7	-5.4	-5.4	-5.2	-5.2	-5.0	-5.3

Table S9. Scoring values obtained with MOE for fisetin per protein-ligand complex of the ensemble

3MXF	3P5O	3U5L	4LYW	4MR4	4NUD	4XYA	4YH3	5KJ0	5M3A	5TI2	5TI3	5U28	5VOM
-6.3	-6.2	-6.3	-6.8	-6.0	-7.0	-7.4	-6.4	-6.4	-7.4	-6.5	-6.5	-6.9	-6.6
-6.3	-6.2	-6.3	-6.8	-6.0	-7.0	-7.0	-6.2	-6.3	-6.6	-6.4	-6.4	-6.9	-6.1
-6.2	-6.0	-6.2	-6.4	-5.9	-7.0	-6.7	-6.2	-6.2	-6.6	-6.4	-6.4	-6.5	-5.9
-6.0	-5.9	-6.2	-6.2	-5.8	-7.0	-6.7	-6.1	-5.9	-6.6	-6.3	-6.4	-6.4	-5.9
-5.9	-5.9	-6.2	-6.1	-5.8	-6.8	-6.7	-6.0	-5.9	-6.5	-6.2	-6.4	-6.3	-5.9
-5.8	-5.9	-6.0	-6.0	-5.8	-6.8	-6.6	-5.9	-5.8	-6.5	-6.1	-6.3	-6.2	-5.9
-5.8	-5.7	-6.0	-6.0	-5.7	-6.7	-6.6	-5.9	-5.7	-6.5	-6.1	-6.2	-6.1	-5.7
-5.8	-5.7	-5.8	-6.0	-5.6	-6.7	-6.6	-5.9	-5.7	-6.4	-6.1	-6.1	-6.1	-5.6
-5.8	-5.7	-5.9	-5.8	-5.6	-6.6	-6.6	-5.9	-5.7	-5.8	-6.0	-6.1	-6.0	-5.6

Table S10. Scoring values obtained with PLANTS for fisetin per protein-ligand complex of the ensemble

3MXF	3P5O	3U5L	4LYW	4MR4	4NUD	4XYA	4YH3	5KJ0	5M3A	5TI2	5TI3	5U28	5VOM
-74.7	-76.1	-76.5	-76.1	-76.6	-76.8	-76.0	-74.3	-72.6	-75.2	-74.6	-79.6	-75.9	-71.6
-73.7	-74.5	-75.3	-74.9	-75.4	-76.3	-73.4	-73.0	-72.3	-74.2	-74.2	-75.2	-73.9	-69.8
-71.9	-73.2	-73.3	-72.3	-73.5	-74.9	-72.3	-71.2	-70.3	-73.4	-73.1	-74.8	-70.7	-68.9
-71.8	-72.0	-73.2	-72.1	-72.6	-73.4	-70.1	-70.9	-69.8	-73.2	-71.7	-72.2	-70.5	-68.7
-71.7	-71.8	-71.6	-71.4	-71.6	-69.3	-69.9	-69.9	-68.9	-73.0	-69.0	-71.6	-70.4	-67.1
-69.6	-70.9	-69.9	-68.0	-71.5	-68.9	-69.3	-69.0	-68.9	-71.2	-68.8	-71.1	-69.2	-66.2
-68.8	-70.8	-69.6	-67.6	-71.2	-68.8	-69.2	-68.9	-67.9	-68.8	-67.9	-70.8	-69.0	-66.1
-68.0	-69.2	-67.2	-66.7	-69.8	-68.6	-69.0	-68.9	-67.0	-68.7	-67.5	-70.0	-66.4	-65.9
-67.8	-68.9	-67.2	-66.5	-69.8	-68.2	-68.7	-66.8	-66.5	-68.1	-67.0	-69.7	-66.2	-65.0

Table S11. Scoring values obtained with Vina for fisetin per protein-ligand complex of the ensemble

3MXF	3P5O	3U5L	4LYW	4MR4	4NUD	4XYA	4YH3	5KJ0	5M3A	5TI2	5TI3	5U28	5VOM
-8.0	-8.1	-8.3	-8.2	-8.4	-8.4	-8.4	-8.2	-8.5	-8.6	-8.5	-8.5	-8.0	-7.9
-8.0	-8.1	-8.3	-8.0	-8.1	-8.3	-8.3	-7.9	-8.5	-8.6	-8.1	-8.5	-7.8	-7.7
-8.0	-8.0	-8.2	-7.9	-8.0	-8.3	-8.3	-7.8	-8.3	-8.5	-8.1	-8.5	-7.7	-7.7
-8.0	-8.0	-8.2	-7.9	-7.8	-8.2	-8.3	-7.8	-8.3	-8.3	-7.9	-8.4	-7.6	-7.7
-7.9	-8.0	-8.1	-7.9	-7.7	-8.2	-8.2	-7.7	-8.1	-8.3	-7.8	-8.2	-7.5	-7.6
-7.8	-7.9	-8.0	-7.8	-7.7	-8.0	-8.2	-7.6	-8.1	-8.2	-7.8	-8.0	-7.5	-7.6
-7.8	-7.6	-7.9	-7.8	-7.6	-7.9	-8.1	-7.4	-8.0	-8.1	-7.8	-8.0	-7.5	-7.4
-7.7	-7.6	-7.8	-7.8	-7.6	-7.7	-8.1	-7.4	-8.0	-8.1	-7.7	-7.9	-7.5	-7.4
-7.5	-7.5	-7.7	-7.6	-7.5	-7.7	-8.1	-7.2	-8.0	-8.1	-7.6	-7.9	-7.4	-7.1

Figure S1. Relaxation protocol and MD workflow used in this work.

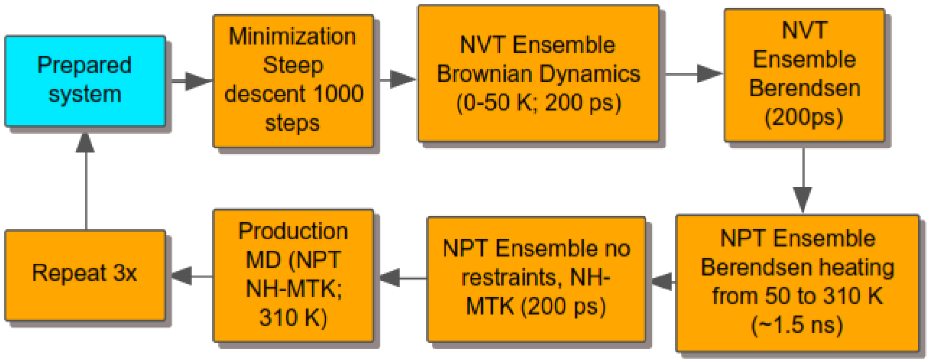

Figure S2. Simulation Quality parameters for the BRD4 protein for 100 ns

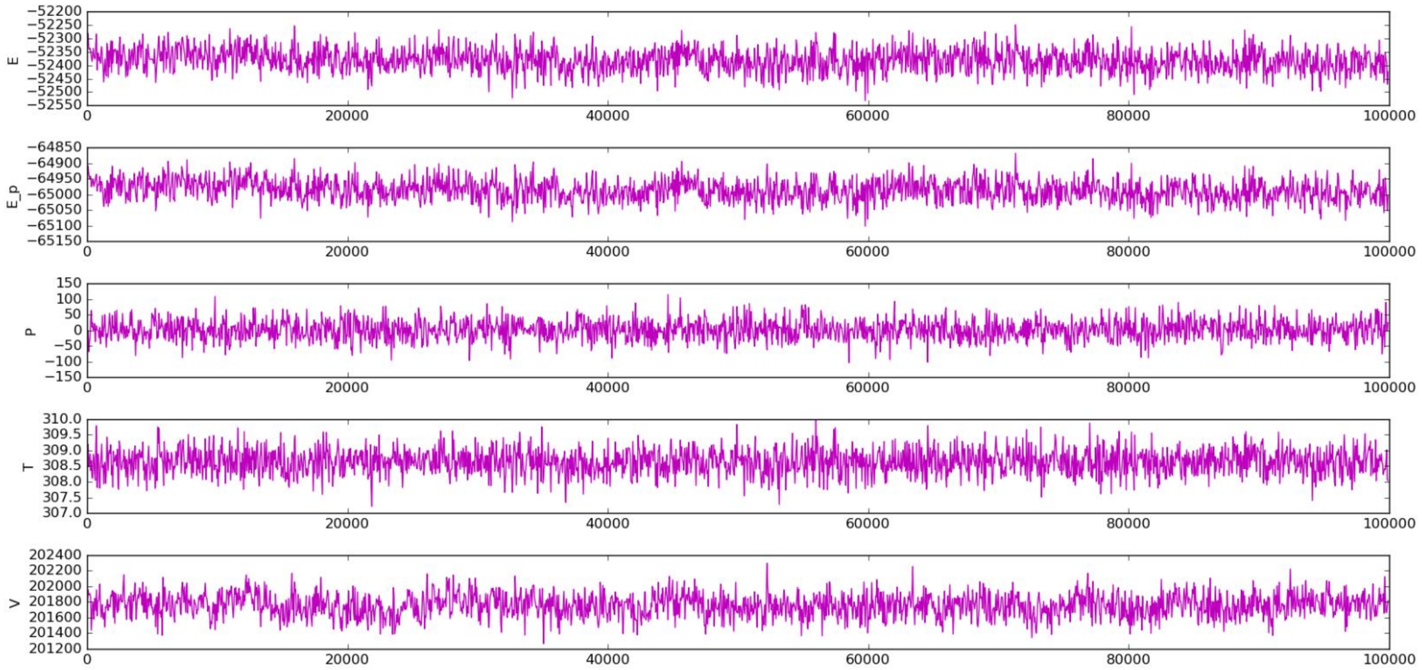

Table S12. Summary values for quality measures for the BRD4 protein

Measure	Average	St. Dev.	Slope (ps^{-1})
Energy (kcal/mol)	**-52381.523**	**43.182**	**0**
E_pot (kcal/mol)	**-64986.594**	**34.152**	**0**
Temperature (K)	**308.638**	**0.405**	**0**
Pressure (bar)	**1.386**	**31.871**	**0**
Volume (Å^3)	**201756.219**	**148.561**	**0**

Figure S3. RMSD values for BRD4 protein for 100ns

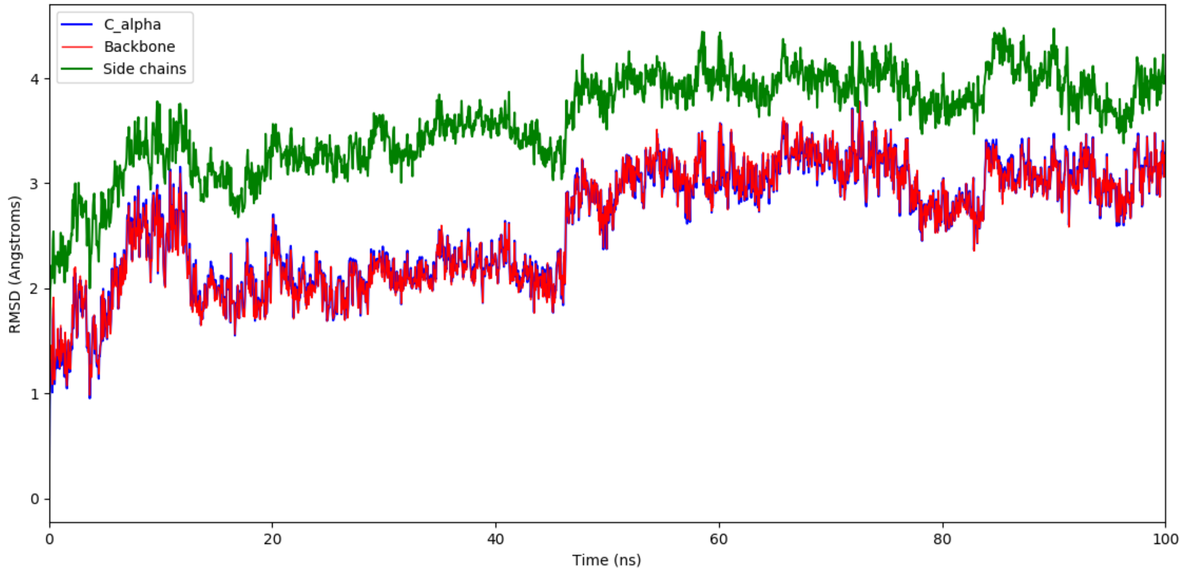

Figure S4. Simulation Quality parameters for the BRD4 protein with fisetine for 100 ns

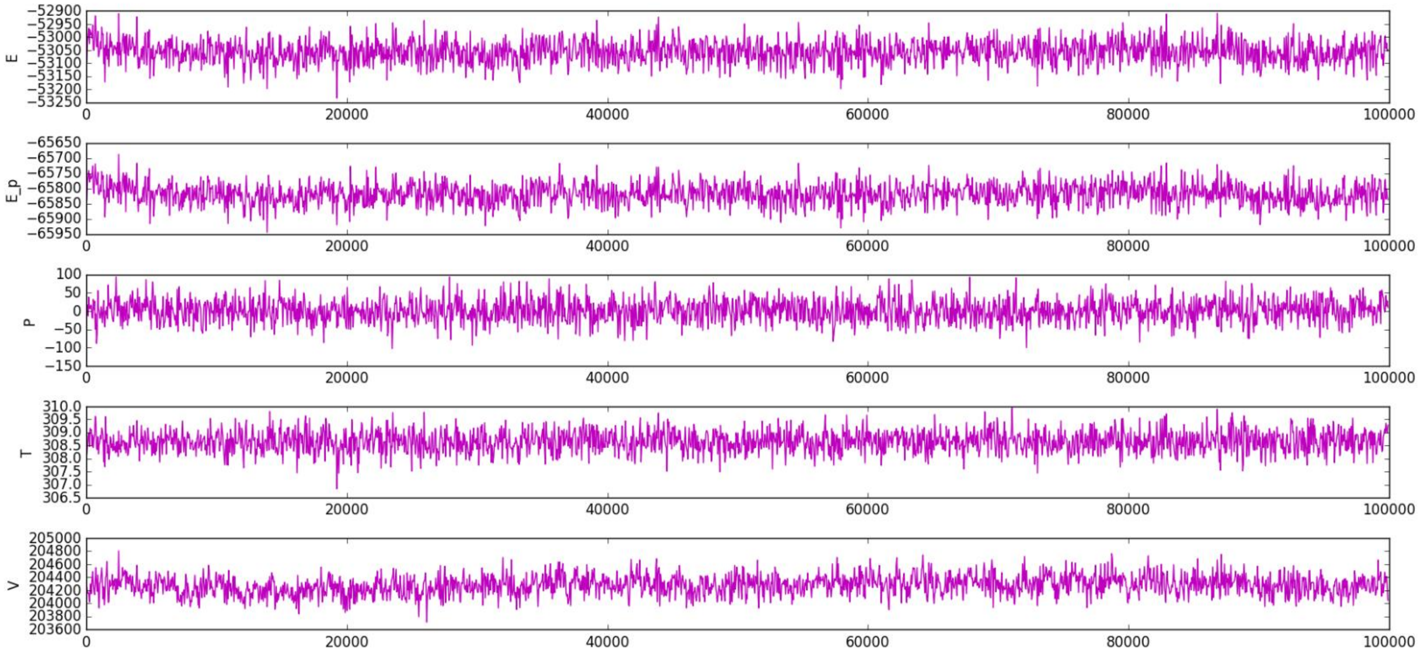

Table S13. Summary values for quality measures BRD4 protein with fisetine

Measure	Average	St. Dev.	Slope (ps^{-1})
Energy (kcal/mol)	**-52181.24**	**44.07**	**0**
E_pot (kcal/mol)	**-65818.84**	**34.67**	**0**
Temperature (K)	**308.652**	**0.412**	**0**
Pressure (bar)	**0.75**	**30.571**	**0**
Volume (Å^3)	**204276.453**	**151.85**	**0.001**

Figure S5. Simulation Quality parameters for the BRD4 protein with amentoflavone for 100 ns

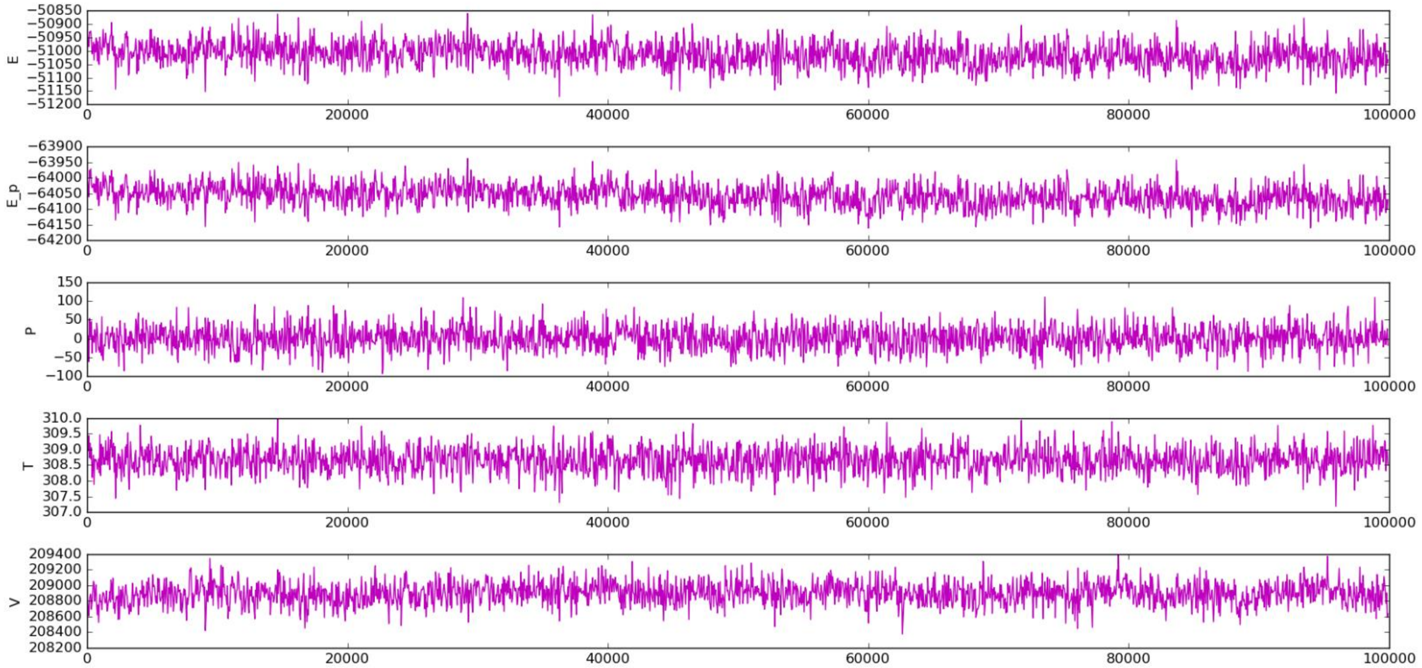

Table S14. Summary values for quality measures BRD4 protein with amentoflavone

Measure	Average	St. Dev.	Slope (ps^{-1})
Energy (kcal/mol)	**-51014.1**	**45.84**	**0**
E_pot (kcal/mol)	**-64057.84**	**35.95**	**0**
Temperature (K)	**308.655**	**0.4**	**0**
Pressure (bar)	**0.946**	**31.592**	**0**
Volume ($Å^3$)	**208895.878**	**142.446**	**0**

Figure S6. Secondary structure of BRD4 as observed for 100 ns. A) BRD4 without ligand. B) amentoflavone. C) fisetin

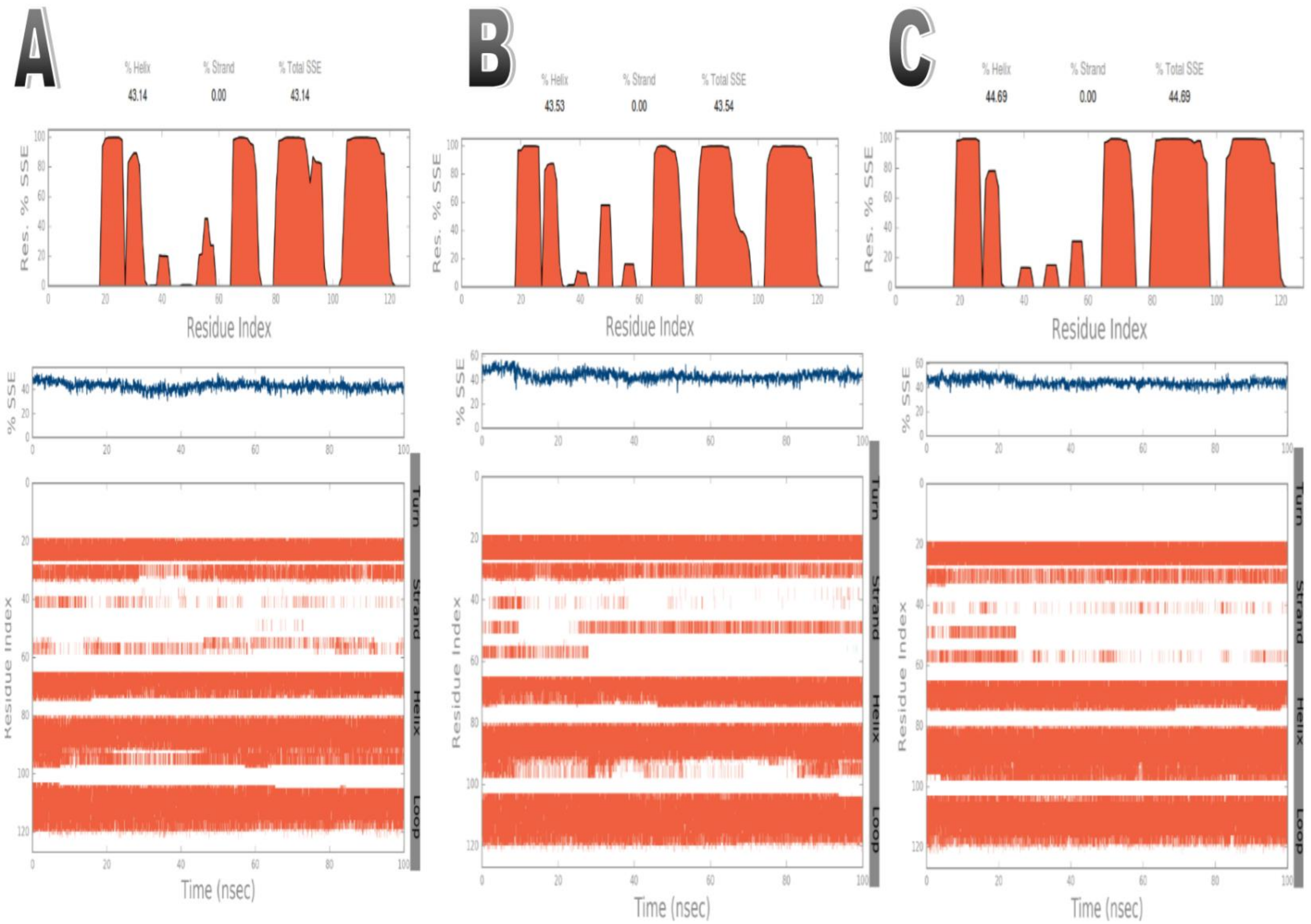

Supplement: Supplementary file 1 [file biomolecules-08-00061-s001.pdf]
